# Supplementary material for: Chromothripsis during telomere crisis is independent of NHEJ, and consistent with a replicative origin
Source: Genome Res. 2019 May;29(5):737–49. doi: 10.1101/gr.240705.118 (PMC6499312; doi:10.1101/gr.240705.118)
Supplement: Supplemental Material [file supp_gr.240705.118_Supplemental_file_1.zip › contigs/annotated_contigs/DB79/contig.2.DB79_length_409_mean_cov_4.89486552567.docx]

**DB79_length_409_mean_cov_4.89486552567**

```
ATTTTTTAAACTTGTATTTGTAATCTTAGAAAATGTGGGCAGTGGGGCTGGGCATGGTGGCTCACACCTGTAATCCCAGCACTTTGGGA
 >chr9:132479648-132479831 + E=2e-94 p=3e-02
GGCCGAGGCGGGCAGATTGCTCGAGGCCAAGAGTTCGAGACCAGCCTGGCCAACATGGTGAAACTCTGTTTCTACTAAAAATACAAAAA

TTAGC|A|CCTTAAAAATCAAATTTTTTTGAGATTATATTATTTCTAATTGTTAAGGTGGTAGAAATCGTATATGAGCATGTACTTTAT
      >chr2:14166694-14166920 - E=1e-121
ACTTATATTTTATTTTTCCTTAGAATAAATTCCTGTAAACTGACTTGATGGGTCAAAATTAGTAAAGTTATTAAGTTTCTTTACTAACA

AAATCCTATTTGTATGTTTATATATATGAAAAATTTACAACTATTCATAATCCTA
```
